# Commentary: Echocardiographic Evaluation of Pulmonary Pressures and Right Ventricular Function after Pediatric Cardiac Surgery: A Simple Approach for the Intensivist

**DOI:** 10.3389/fped.2018.00136

**Published:** 2018-05-08

**Authors:** Stefan Kurath-Koller, Sabrina Schweintzger, Martin Köestenberger

**Affiliations:** Division of Pediatric Cardiology, Department of Pediatrics, Medical University Graz, Graz, Austria

**Keywords:** PAH, pediatrics, RV function, echocardiography, critical care

A recent multicenter analysis in pediatric intensive care units shows, that despite of therapeutic advances, the disease burden and mortality of children with pulmonary arterial hypertension (PAH) remains significant (1). This calls into attention a recent Review by in Frontiers in Pediatrics which we read with great interest. The article “Echocardiographic Evaluation of Pulmonary Pressures and Right Ventricular Function after Pediatric Cardiac Surgery: A Simple Approach for the Intensivist” by Beghetti (2) describes an extremely useful overview how to use an echocardiography approach to quickly evaluate pulmonary arterial pressure (PAP) and right ventricular (RV) function in children in regard of PAH in the intensive care unit setting. The author elaborates that in this population there is a clear need for a thoroughly evaluation of children with an “RV under pressure,” using the first line investigation echocardiography (2). Children with PAH still are challenging in regard of evaluation and treatment (3).

This land marking echocardiographic approach of Beghetti uses direct and indirect echocardiographic measurements including the commonly used tricuspid regurgitant (TR) jet velocity, pulmonary regurgitation (PR) jet (used to calculate the mean PAP and diastolic PAP), D-shaping of the left ventricle (LV), the LV eccentricity index, right ventricular fractional area change (RVFAC), tricuspid annular plane systolic excursion (TAPSE), and tissue Doppler imaging (TDI) parameters. This approach is in parts in agreement with recent US and European pediatric PAH guidelines (4–6). To date, in our intensive care unit (ICU) we use calculation of the TR jet, the PR jet, the pulmonary artery acceleration time (PAAT), the tricuspid annular peak systolic velocity (TDI), and the tricuspid annular plane systolic excursion (TAPSE) measurements to quickly investigate RV function and pressure in postoperative patients. We completely agree with the author (2) that pulmonary vascular resistance (PVR) is too complex to measure in critical care medicine when a quick evaluation is needed and furthermore that more detailed/complex measurements such as TEI index, strain measurements, and 3D measurements are way too time consuming in this specific setting.

Due to the convincing approach of Maurice Beghetti will from now follow this approach in our ICU.

Currently there is an ongoing discussion on the indication/need for cardiac catheterization in children with suspected PAH (7). If properly performed, echocardiography may make cardiac catheterization expandable in the immediate postoperative period (7), taking into account the critical condition of these patients and potential complications (8).However, cardiac catheterization still remains the gold standard for the diagnosis of a PAH (8). In addition we agree with the notice of Beghetti (2) that more data of treatment strategies for pediatric PAH, although partially existing (9, 10), are highly recommended.

We want to thank the author for addressing the need for quick, simple, and reproducible measurements in children with suspected PAH and/or RV dysfunction for the intensivist and want to highlight his article, which now provides essential tools to acquire this target (2). It would be of benefit for the audience of Frontiers in Pediatrics if doctor Beghetti would be willing to provide a simple Table/Figure with the few variables to quickly measure RV function and PAH estimation—as a short survey for intensivists all over the world. With this remarkable approach, we are convinced that in near future the evaluation and decision making on potential PAH or RV dysfunction therapy will be more easily feasible in postoperative congenital heart disease children. Beyond this, we recommend this approach to every ICU physician.

## Author contributions

All authors listed have made a substantial, direct and intellectual contribution to the work, and approved it for publication.

### Conflict of interest statement

The authors declare that the research was conducted in the absence of any commercial or financial relationships that could be construed as a potential conflict of interest.
